# Short-term influences of radiation on musculofascial healing in a laparotomy rat model

**DOI:** 10.1038/s41598-019-48201-5

**Published:** 2019-08-15

**Authors:** Youbai Chen, Qixu Zhang, Yewen Wu, Cynthia D. Branch-Brooks, Charles E. Butler

**Affiliations:** 1 Department of Plastic Surgery, The University of Texas MD Anderson Cancer Center, Houston, TX 77030 USA; 2 Department of Plastic and Reconstructive Surgery, First Medical Center, Chinese PLA General Hospital, Beijing, 100853 China

**Keywords:** Experimental models of disease, Preclinical research

## Abstract

Preoperative radiation is associated with an increased risk of wound complications. However, the influences of radiation on musculofascial wound healing remains unclear. The purpose of the study was to investigate the short-term effects of preoperative local radiation on the musculofascial healing of laparotomy incisions in a rat model. Eighteen Fischer 344 rats received radiation doses of 0, 10, or 20 Gy to the abdominal wall and underwent laparotomy 4 weeks later. Two weeks after laparotomy, samples of irradiated muscle were harvested for mechanical tests, histological (Hematoxylin & Eosin, and Masson’s Trichrome) and immunohistochemical analyses using KI67, CD31, TGF-β, and MYOD1 antibodies. The elastic modulus (EM), maximum strain (MS), and ultimate tensile strength (UTS) in the 20-Gy group were significantly weaker than those in the 0-Gy group. The EM and UTS in the 20-Gy group were significantly lower than those in the 10-Gy group. The UTS and MS in the 10-Gy group were significantly lower than those in the 0-Gy group. The mean number of inflammatory cells per mm^2^ in the 20-Gy group was significantly larger than those in the 10- and 0-Gy groups. The mean numbers of CD31-, KI67-, and MYOD1-positive cells, the optical density of TGF-β, and the microvessel density in the 20-Gy group were significantly smaller than those in the 10- and 0-Gy groups. These results indicated that radiation delays musculofascial healing and decreases mechanical strength of the laparotomy incision by creating a chronic inflammatory environment, inhibiting cell proliferation, angiogenesis, granulation maturation, collagen deposition, and muscular regeneration in a dose-dependent manner. The impaired biomechanical, histological and molecular properties may be associated with the higher risk of wound complications in patients who undergo radiotherapy prior to laparotomy.

## Introduction

Wound healing is a complicated process that includes inflammation, proliferation, regeneration, and remodeling, involving interactions among a variety of cells, chemokines, cytokines, growth factors, and extracellular matrix^1^. Radiotherapy plays a critical curative role in the comprehensive treatment of a broad spectrum of cancers owing to its benefits such as reducing tumor volume, controlling tumor progression and recurrence, increasing overall survival rates. Clinically, it is not uncommon to perform surgery on a previously irradiated field. Radiation may disturb the above mentioned stages and interactions in wound healing process, increase the risk of wound complications including delayed healing, infection, ulceration, erosion, necrosis, dehiscence, and fibrosis^2–5^. For example, radiotherapy of abdominal malignancies directly affects the abdominal wall, inevitably affects the integrity of abdominal wall structures and may adversely affect the outcome of abdominal wall reconstruction after oncologic resection. More than 350,000 abdominal wall reconstructions are performed annually in the United States to repair oncologic defects and hernias^6^. A number of challenges with post-radiotherapy abdominal wall reconstruction have been described including difficulty in distinguishing anatomic planes, extensive soft tissue fibrosis, reduced tissue pliability, mobilization of the abdominal wall, and prolonged healing time^7–9^. Incisional hernia, one of the most significant challenges after abdominal wall surgery, occurs most frequently through previous incision or scar within one year postoperatively with a high incidence of 10–20% and recurrence rate up to 20%^6^. To avoid wound complications in irradiated abdomens, abdominal wall reconstruction following radiotherapy often requires a well-vascularized flap or component separation.

The potential mechanism of radiation’s impact on skin wound healing has been extensively investigated^10–12^. Wang *et al*.^13^ showed that radiation impaired vascular structure and decreased vessel density, leading to poor microcirculation, hypoxia, and insufficient nutrition. Post-radiation skin wounds are associated with a significant decrease in burst strength. Furthermore, inflammation infiltration and fibroblast migration from vessels around the wound were reduced because of the compromised vasculature and circulation. These alterations caused abnormal granulation tissue formation and collagen deposition^14^. However, compared with abundant studies in skin wound healing, few studies have investigated the post-radiation musculofascial healing of a laparotomy. The knowledge gap of short-term influences of radiation on the musculofascial healing needs to be elucidated to optimize radiotherapy and surgery plans for effective prevention of incisional hernia and other wound complications.

The purpose of this study is to investigate the impact of radiation on the musculofascial healing of a laparotomy incision in a rat model and to explore the biomechanical and histopathological change in post-radiation musculofascial incisional healing. We hypothesized that radiation would delay muscle wound healing and decrease mechanical strength of the incision in a dose-dependent manner. We tested our hypothesis through the following specific aims: (1) to develop a post-radiation laparotomy rat model; (2) to compare the biomechanical, histopathological and molecular properties of laparotomy incisions exposed to no radiation, 10-Gy, and 20-Gy radiation.

## Results

### Gross observation and adhesions

All rats tolerated the radiation treatment well without radiation-related complications or apparent changes in behavior. One out of 6 rats in the 20-Gy group had slight epilation 12 days after radiation. Within the first 3 days after laparotomy, the rats were extremely fatigued with decreased activity, appetite and weight (average 301 ± 23 g on the third day after laparotomy). All wounds healed well without wound complications. One out of 6 rats in the 10-Gy group and 1 out of 6 rats in the 20-Gy group had greater omentum adhesions but no intestinal adhesions.

### Mechanical tests

**Figure 1:**
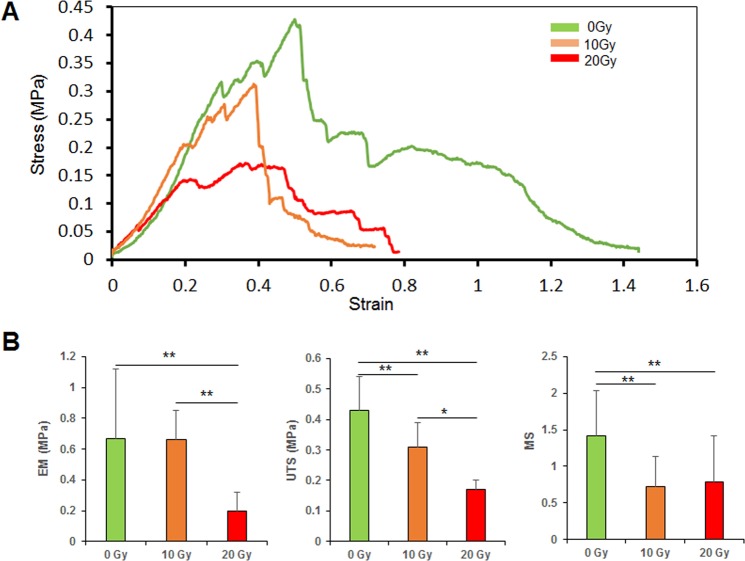
Mechanical testing results. (**A**) Strain-stress curves of muscle samples from the 0-, 10-, and 20-Gy groups. (**B**) EM, UTS, and MS of the 0-, 10-, and 20-Gy groups (n = 6 per group). UTS, ultimate tensile strength; MS, maximum strain; EM, elastic modulus. **p < 0.01, *p < 0.05.

The stress-strain curves and comparisons of the UTS, MS, and EM among the 0-, 10-, and 20-Gy groups are illustrated in Fig. 1. The UTS (0.43 ± 0.11 MPa), MS (1.42 ± 0.62), and EM (0.67 ± 0.45 MPa) of the 0-Gy group were significantly higher than those of the 20-Gy group (UTS, 0.17 ± 0.03 MPa; MS, 0.79 ± 0.63; EM, 0.2 ± 0.12 MPa). In addition, the UTS (0.31 ± 0.08 MPa) and MS (0.72 ± 0.41) of the 10-Gy group were significantly lower than those of the 0 Gy group; however, the UTS and EM (0.66 ± 0.19 MPa) of the 10-Gy group were significantly higher than those of the 20-Gy group.

### Histochemical analysis

**Figure 2:**
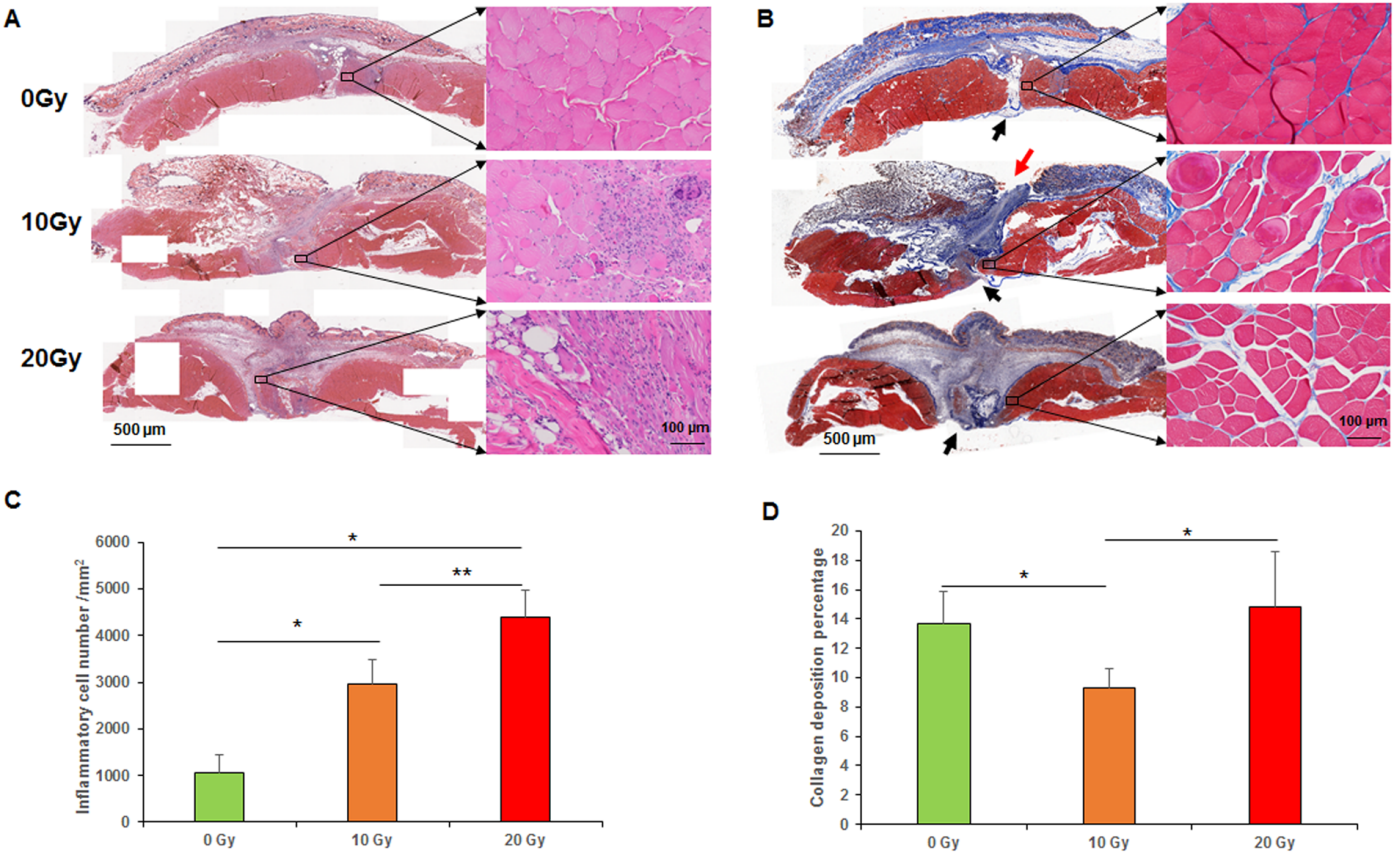
Histochemical analyses. (**A**) H&E staining. (**B**) Masson trichrome staining (arrows indicated wound incisions). (**C**) Mean numbers of inflammatory cells per mm^2^. (**D**) The overall collagen deposition ratio of the panoramic cross-section. **p < 0.01, *p < 0.05.

The results of the histochemical analyses are illustrated in Fig. 2. The 0-Gy group had distinct epidermis, dermis, subcutaneous fat, and muscle. The epidermis was completely healed with slight thickening in the 0-Gy group but was unhealed in the 10-Gy group. The mean numbers of inflammatory cells in the 0-, 10-, and 20-Gy groups were 1059.2 ± 397.6, 2969.6 ± 508.8, and 4386.4 ± 572.8 per mm^2^, respectively. The 0-Gy group had fewer inflammatory cells than 10- and 20-Gy groups (*p* < 0.05, the 0-Gy group compared with 10- and 20-Gy groups; *p* < 0.01, the 10-Gy group compared with the 20-Gy group) (Fig. 2C). The 0-Gy group had few inflammatory cells in granulation tissue, whereas the 10- and 20-Gy groups had excessive inflammatory cell infiltration. In the 10- and 20-Gy groups, the gap between the incision margins was filled with immature granulation tissue incorporating large numbers of inflammatory cells and fibroblasts, whereas, in the 0-Gy group, the gap was filled with mature granulation and fibrous connective tissue. More importantly, the laparotomy incisions in the 0-Gy group were nearly healed (Fig. 2A).

The collagen area ratios in the 0-, 10-, and 20-Gy groups were 13.65%, 9.27%, and 14.85%, respectively (*p* < 0.05, 0-Gy group compared with 10-Gy group, 10-Gy group compared with 20-Gy group) (Fig. 2D). Although the 20-Gy group had a higher collagen area ratio, its mechanical strength was less than that of the 0-Gy group, likely because the collagen fibers in the immature granulation tissue were loose and disorganized. In contrast, the collagen fibers in the 0-Gy group were aligned in parallel and sufficiently well organized to enhance mechanical properties, which was consistent with our mechanical test results. Myocytes in the 0-Gy group showed normal morphology without noticeable atrophy or abnormal intercellular space, whereas myocytes around the incision margins in the 20-Gy group showed increased nuclei size, atrophy, swelling, degeneration, necrosis, and intercellular space (Fig. 2B).

### Immunohistochemical analysis

**Figure 3:**
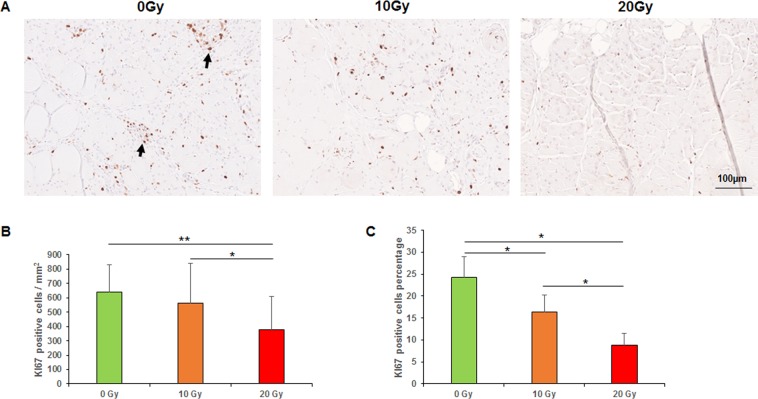
Immunohistochemical analyses of KI67. (**A**) KI67 staining (arrows indicated positive staining). (**B**) Mean numbers of KI67-positive cells per mm^2^. (**C**) Percentages of KI67-positive cells. **p < 0.01, *p < 0.05.

**Figure 4:**
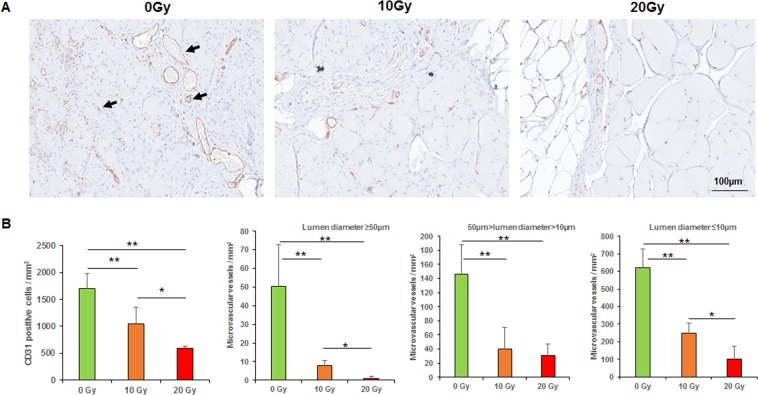
Immunohistochemical analyses of CD31. (**A**) CD31 staining (arrows indicated positive staining). (**B**) Mean numbers of CD31-positive cells per mm^2^. (**C**) Mean numbers of different lumen diameter size of microvascular vessels per mm^2^. **p < 0.01, *p < 0.05.

**Figure 5:**
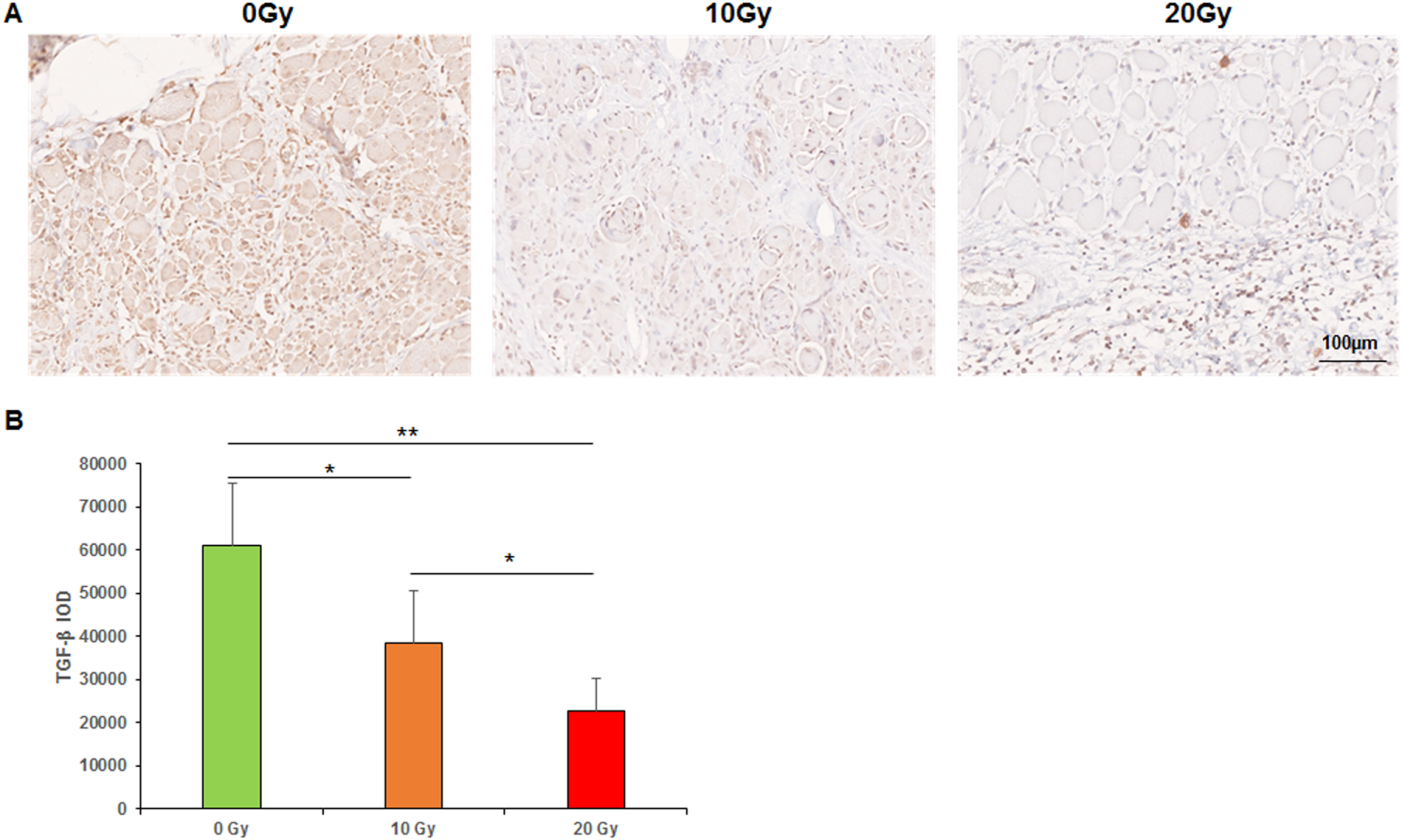
Immunohistochemical analyses of TGF-β. (**A**) TGF-β staining. (**B**) Mean IOD of TGF-β. IOD: Integral optical density. **p < 0.01, *p < 0.05.

**Figure 6:**
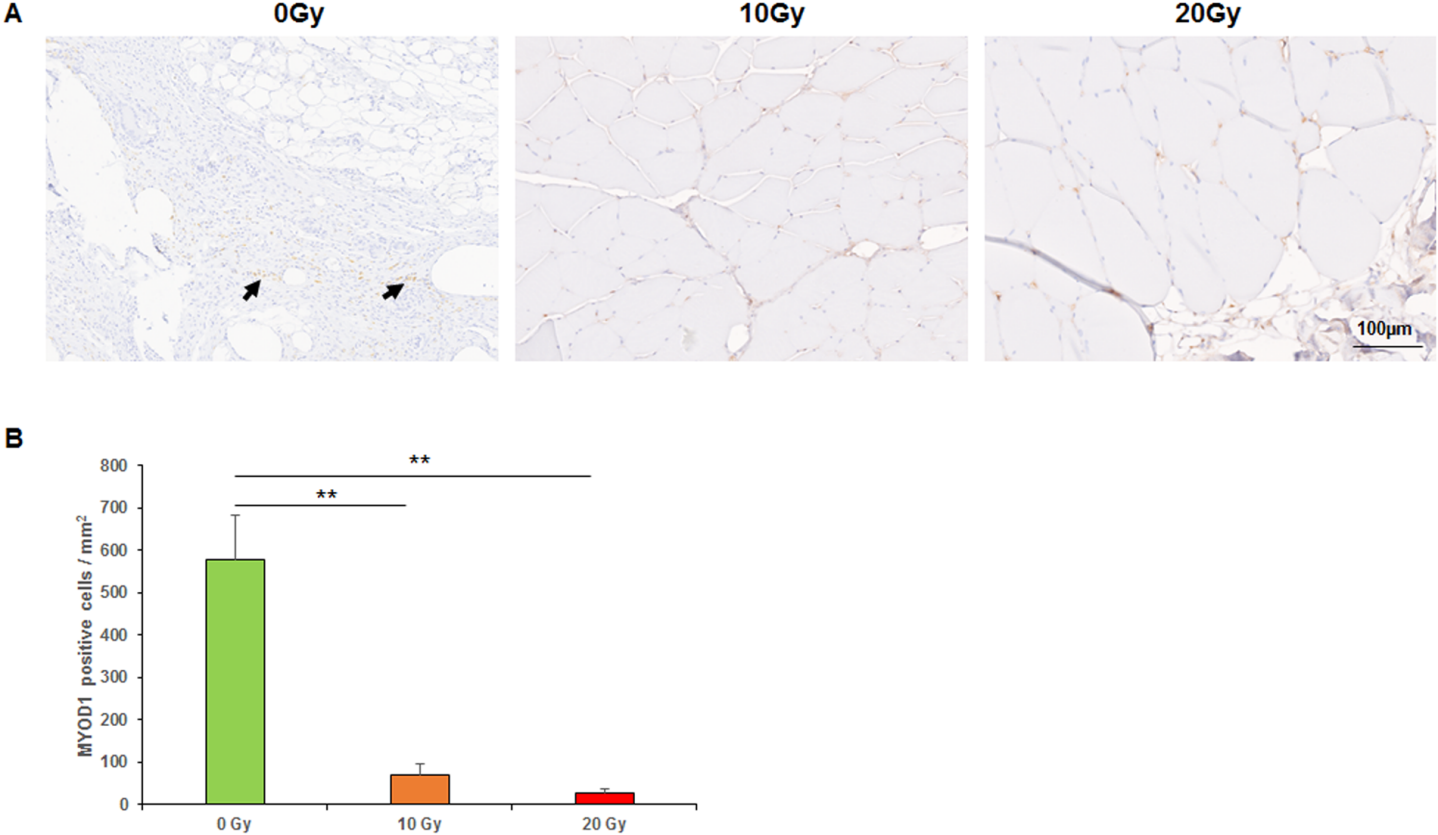
Immunohistochemical analyses of MYOD1. (**A**) MYOD1 staining (arrows indicated positive staining). (**B**) Mean numbers of MYOD1-positive cells per mm^2^. **p < 0.01, *p < 0.05.

The results of the immunohistochemical analyses are illustrated in Figs 3–6. We observed a high expression of KI67 in the basal layer of the epidermis and the cutaneous appendages, as well as in the granulation tissue; a medium expression in muscle satellite cells at the incision margins and almost no expression in normal muscle away from the incision. The mean numbers of KI67-positive cells per mm^2^ in the 0-, 10-, and 20-Gy groups were 639.2 ± 191.2, 562.4 ± 280.8, and 380 ± 227.2, respectively (*p* < 0.01, the 0-Gy group compared with the 20-Gy group; *p* < 0.05, the 10-Gy group compared with the 20-Gy group). The percentages of KI67-positive cells in the 0-, 10-, and 20-Gy groups were 24.3%, 16.3%, and 8.8%, respectively (p < 0.05; Fig. 3).

The mean number of CD31-positive cells per mm^2^ in the 0-Gy group (1700 ± 287.2) was significantly higher than those in the 10-Gy group (1051.2 ± 307.2) and 20-Gy group (588.8 ± 41.6; *p* < 0.01, the 0-Gy group compared with 10- or 20-Gy group; *p* < 0.05, the 10-Gy group compared with the 20-Gy group). The microvessel density per mm^2^ of the 0-Gy group (lumen diameter [LD] > 50 μm, 50.4 ± 22.4; 50 μm ≥ LD ≥ 10 μm, 146.4 ± 41.6; LD < 10 μm, 620.8 ± 106.4) was higher than those of the 10-Gy group (8 ± 2.4, 40 ± 30.4, 247.2 ± 58.4) and 20-Gy group (0, 30.4 ± 16.8, 100.8 ± 75.2; *p* < 0.01, 0-Gy group compared with 10-Gy group; *p* < 0.05, 10-Gy group compared with 20-Gy group). The density of microvessels with any LD dramatically declined as the radiation dose increased (Fig. 4).

The mean integral optical densities (IOD) of TGFβ in the 0-, 10-, and 20-Gy groups, were 61215.0 ± 14327.9, 38591.3 ± 12076.1, and 22754.0 ± 7534.9, respectively (*p* < 0.05, the 0-Gy group compared with the 10-Gy group, the 10-Gy group compared with the 20-Gy group; *p* < 0.01, the 0-Gy group compared with the 20-Gy group; Fig. 5).

MYOD1 expression was only detected in granulation tissue and muscle tissue around the incision margins. The numbers of MYOD1-positive cells in the 0-, 10-, and 20-Gy groups were 578.2 ± 104.8, 69.6 ± 26.4, and 28 ± 8.4 per mm^2^, respectively (*p* < 0.01, the 0-Gy group compared with the 10-Gy group, the 0-Gy group compared with the 20-Gy group; Fig. 6).

## Discussion

Radiation initiates a cascade of molecular events that have profound effects on wound healing. The results of present study tested our hypothesis that radiation impaired the musculofascial healing and decreased the biomechanical strength of the laparotomy incision by creating a chronic inflammatory environment, inhibiting cell proliferation, angiogenesis, granulation tissue maturation, collagen deposition/remodeling, and muscular regeneration in a dose-dependent manner.

### Model

Many clinical studies have reported that preoperative radiation is associated with a higher risk of wound complications after various surgeries^15,16^. The effect of radiation on the wound healing process depends on the sequence of radiation and surgery, the interval between radiation and surgery, and the type and dose of the radiation^17^. Previous study showed that the adverse effect of radiation on wound healing was greater when irradiation was performed before surgery than when it was performed after surgery^18^. Preoperative irradiation is usually assumed to have a therapeutic ratio (tumor control relative to the risk of long-term radiation-induced complications) superior to that of postoperative irradiation. Although the optimum timing of surgery following preoperative radiation therapy is still controversial, surgery is typically performed 3–6 weeks after radiotherapy, when an acute radiation-induced injury has subsided and delayed damage has not occurred^19^. This provides a relatively stable environment for wound healing. In our study, laparotomy was performed 4 weeks after radiation to simulate current clinical scenarios maximally. Gamma and X-rays were the most commonly used types of radiation clinically and experimentally^14,20,21^. We used radiation generated by a XRAD 225Cx source because the depth of radiation could be limited to 1 cm in case of excessive damage to abdominal viscera. Local X-ray with doses >35 Gy could induce severe radiation injury. A dose up to 20-Gy is sufficient to investigate the short-term side effect of radiation.

### Mechanical properties

Radiation could impair the mechanical properties of biological tissue^19,22^. The healed abdominal wall incision must have adequate mechanical strength to prevent incisional hernia. Previous data was limited regarding the mechanical properties of the post-radiation musculofascial incision. Zhou *et al*. showed an average UTS of 0.21 ± 0.08 MPa, MS of 1.21 ± 0.27, and EM of 0.19 ± 0.07 for native muscle^23^. Our results collectively indicated that radiation reduced the UTS, MS, and EM in a dose-dependent manner, which might be the biomechanical weakness of an incisional hernia in patients with previous radiotherapy and laparotomy. The mechanical strength of muscle samples was too weak to resist tension 2 weeks postoperatively. This agrees with our clinical experience; when implant removal or re-operation through the original muscle incision is needed, the repaired muscle tissue can be easily separated often times with manual tension and without re-incising the wound site.

### Inflammation

Inflammation is the initial phase of wound healing, which usually lasts 2–3 days in a physiological wound healing process. After an acute injury, macrophage infiltration peaks within 48 h. In the absence of a macrophage response, muscle regeneration is absent. On completing their task, inflammatory cells must be reduced from the wound before progression to the next phase of healing proliferation. However, radiation decreased short-term inflammation by impeding local inflammatory cell infiltration but might create a chronic inflammatory environment^24^. We found more inflammatory cells/HPF in 20- and 10-Gy groups compared to the 0-Gy group. At the time of muscle sample harvest (6 weeks after radiation and 2 weeks after laparotomy), the inflammation in the 0-Gy group had subsided. However, inflammation was still ongoing with extensive inflammatory cells in granulation and muscle tissue surrounding the incision in both 10- and 20-Gy groups. This indicated that radiation created a chronic inflammatory microenvironment.

### Proliferation

Radiation affects cell-cycle progression in fibroblasts, leading to apoptosis of fibroblasts. This causes fewer fibroblasts in the wound margins, compromised collagen deposition and remodeling, and insufficient mechanical strength. Previous studies^18,23^ showed the capacity of muscle to resist tension was reduced due to axial misalignment of the collagen fibers, causing reduction of mechanical properties of muscle. Cui, *et al*.^25^ demonstrated chronic inflammation, thinner collagen fibrils and fibers, disorganized collagen, and hyaline fibro degeneration of muscle fibers after radiation. Several studies reported on an imbalance between type I collagen and type III collagen in the rectus sheath, transversalis fascia and skin of patients with both primary and recurrent incisional hernias. Rosch, *et al*. found the abnormal collagen formation was the major cause of the hernia. They found collagen I/III ratio was significantly higher in a recurrent hernia^26^. In accordance with literature and our mechanical test results, we found that the collagen fibers in the 20-Gy group were disorganized, whereas they were aligned in parallel and sufficiently well organized in the O-Gy group.

Cell proliferation is the cornerstone of wound healing. Radiation is thought to prevent satellite cell mitosis by causing breaks in strands of the cell’s DNA. A reduction of proliferation of fibroblasts, satellite cells, and local stem cells was observed in irradiated tissue^27^. Olivé, *et al*.^28^ showed that gamma irradiation affects single skeletal muscle cells (satellite cells or myoblast) during development and induces cell apoptosis. Jurdana, *et al*.^29^ evaluated long-term effects of different doses of radiation on human skeletal muscle myoblast proliferation and stress response capacity. Cells were irradiated with a dose rate of 2 Gy/min with graded doses (2–8 Gy). Their results showed that myoblasts are sensitive to irradiation regarding their proliferation capacity. KI67 known as a specific marker of cell proliferation transiently expressed in the nuclei of proliferative cells. The present study showed a high expression of KI67 in the basal layer of the epidermis and the cutaneous appendages, as well as in the granulation tissue; a medium expression in muscle satellite cells at the incision margins; and almost no expression in normal muscle away from the incision margins. Consistent with the literature, we observed fewer proliferative cells as the dose of radiation increased. This suggests that radiation remarkedly reduced the cell proliferation, leading to delayed collagen production and secretion, myofibroblast differentiation and regeneration.

Angiogenesis is critical to proper wound healing because neovascularization provides sufficient oxygen and nutrition to a wound site, thus establishing a favorable environment for wound healing. We previously reported the impact of radiation on vascularization and angiogenesis^30^. It is widely accepted that radiation directly injures vascular endothelial cells and vascular wall, decreases the capillary number. CD31, also known as platelet endothelial cell adhesion molecule 1, is highly expressed in inter-endothelial cell junctions and thus frequently used as a marker of vascular endothelial cells. Our study showed that 10-Gy or 20-Gy of radiation inhibited vascular endothelial cells and diminished microvessel density, with the 20-Gy dose having the most profound effect on vessel density. This compromised vascularization contributes to reduced microcirculation, depleted perfusion, and reduced nutrition.

TGF-β plays an important role in wound healing by facilitating the formation and maturation of granulation tissue, as well as the synthesis and secretion of collagen and other extracellular matrix components. TGFβ expression is continuously high in normal wound healing. The effect of radiation on TGF-β remains to be an active debate. Some authors reported higher expression of TGF-β in irradiated tissue compared with normal tissue^19^. Gallet, *et al*.^31^ found that the expression of TGF-β substantially increased after radiation. The level of TGF-β was 2.2 times higher than that measured in unirradiated tissue 6 weeks after radiation. We observed a high expression of TGF-β in fibroblasts in the granulation tissue near the incision. Unlike their study, we found lower expression levels of TGF-β in the 10- and 20-Gy groups than in the 0-Gy group. Our results indicated that radiation suppressed TGF-β expression, thereby influencing granulation and collagen remodeling, which is believed to be associated with a decreased mechanical strength of musculofascial incision.

Radiation inhibits muscle regeneration by damaging satellite cells. Previous studies showed the development of satellite cells into regenerating muscle fibers appeared to be hampered after radiation^32,33^. Jurdana, *et al*.^29^ affirms that radiation affects muscle satellite cells, impairing their activation, proliferation and differentiation, as well as interfering in membrane permeability and affecting sodium and potassium pump. MYOD1, the key regulator and switch gene in myogenesis, is a marker of muscle repair and regeneration. MYOD1 activates the transcription of specific myogenic genes and promotes myogenic differentiation. MYOD1 is not expressed in dormant satellite cells but activated satellite cells and other stem cells with myogenic potential. The present study showed that the 10- and 20-Gy groups had significantly fewer MYOD1-positive cells than the 0-Gy group did. This suggested that radiation impaired muscle repair and regeneration by inhibiting MYOD1 expression; however, the exact molecular mechanism requires further research.

### Study limitation and prospective view

Our study was not without potential limitations. Firstly, we did not include a control group with radiation-only because we focused on a common clinical scenario when a laparotomy is performed on a previous irradiated abdomen.

Secondly, the rats were euthanized and muscle samples harvested only 2 weeks after the operation; however, wound healing is a long process that cannot be assessed at a single time point. Clinically, the process of laparotomy healing over time varies significantly depending on the patient’s characteristics, comorbidities, and surgical factors including the suture technique and the mesh-based repair strategy. Uncomplicated laparotomy normally requires 2 to 6 weeks for wound healing, but the time could be considerably prolonged if the patient has comorbidities/complications that compromise wound-healing potential, such as obesity, immunosuppression, pulmonary disease, malnutrition, wound infection, etc.^7^. Although the biologic healing is ongoing over time, the overall strength of the laparotomy incision still remains decreased during the first postoperative week and then starts to increase after 1 week in a normal wound. Obviously, adding another time point (e.g. 2 months after wounding) would help understanding the remodeling phase of post-radiation musculofascial healing.

Thirdly, the current study has been only focused on the radiated laparotomy model validation and short-term morphological changes of musculofascial tissue wound healing. To illustrate molecular mechanism and signaling pathway of radiation’s impairment on musculofascial incisional healing, only histochemical and immunohistochemical evaluation are apparently insufficient, a further study with genomic/protein microarray analysis is warranted. Indeed, a number of studies have investigated the molecular/genes profile of radiated skin tissue. Johnson, *et al*.^34^ reported that, of the 210 proteins detected in a reverse phase protein array (RPPA), fibronectin was the most significantly and consistently downregulated in radiation-damaged skin. Benderitter, *et al*.^35^ found a specific set of genes, i.e. SOD1, GPX1, TDX1, TDX2 and HSP60, implicated in the redox control of normal skin repair after radiation exposure, whereas HOX1 and HOX2 were involved in the pathological skin repair. The authors further concluded that reactive oxygen species (ROS) modulation is a key element of the healing response after cutaneous exposure to radiation and that the collapse of skin antioxidant status interferes directly with wound healing in skin after radiation exposure. Using a miRNA microarray analysis, among 890 differentially expressed miRNAs identified, Islam, *et al*.^36^ validated two major miRNA seed sequences (miR-690 and miR-223) are specifically associated with skin damage in a radiation combined skin-burn trauma mice model. Considering all of that information together, radiation causes direct cellular DNA damage by strand breaks and indirect damage by free radicals, consequently leading to blood vessels impairment, hypoxia, abnormal inflammatory response, stromal cell (e.g. fibroblasts) dysfunction, and aberrant collagen deposition/remodeling. These alterations collaboratively delay skin wound healing and increase the risk of skin wound complications^10–13^. The knowledge regarding the effect of radiation on skin wound healing based on current genes expression/proteins profile data may shed some lights on the putative changes of the post-radiation musculofascial healing.

On the other hand, there are many clinical predictor variables involving the association between preoperative radiotherapy and post-laparotomy hernia^37^. Despite the similarity to human, the cellular and molecular process during post-radiation in a rat model might not be identical. Our results should be extrapolated to humans with caution.

## Conclusions

We successfully developed a radiation-compromised laparotomy model. We found that radiation delayed the healing of the musculofascial incision and decreased the biomechanical strength by creating a chronic inflammatory environment that reduced angiogenesis, delayed collagen deposition and remodeling, impeded granulation mature, suppressed cell proliferation, and inhibited muscular regeneration in a dose-dependent manner. The impaired biomechanical, histological and molecular properties may be associated with the higher risk of wound complications in patients who undergo combined radiotherapy and laparotomy. This model lays the foundation to elucidate the cellular and molecular mechanisms that irradiation impaired muscle wounds so that rational therapeutic strategies can be developed to improve muscle wound healing and/or prevent hernia formation and other wound healing complications.

## Materials and Methods

### Animals

All protocols were approved by the Institutional Animal Care and Use Committee of The University of Texas MD Anderson Cancer Center and met the requirements of the Animal Welfare Act (ACUF#00000424-RN02). Eighteen male Fischer-344 rats (Charles River Laboratories, Wilmington, MA) with a mean age of 3 months and weight of 308 ± 31 g, were housed in separate pathogen-free cages with unlimited access to standard laboratory food and drinking water with controlled temperature, humidity and an automatically regulated 12-h light/dark cycle for 2 weeks.

### Radiation, surgery, and sample harvest

**Figure 7:**
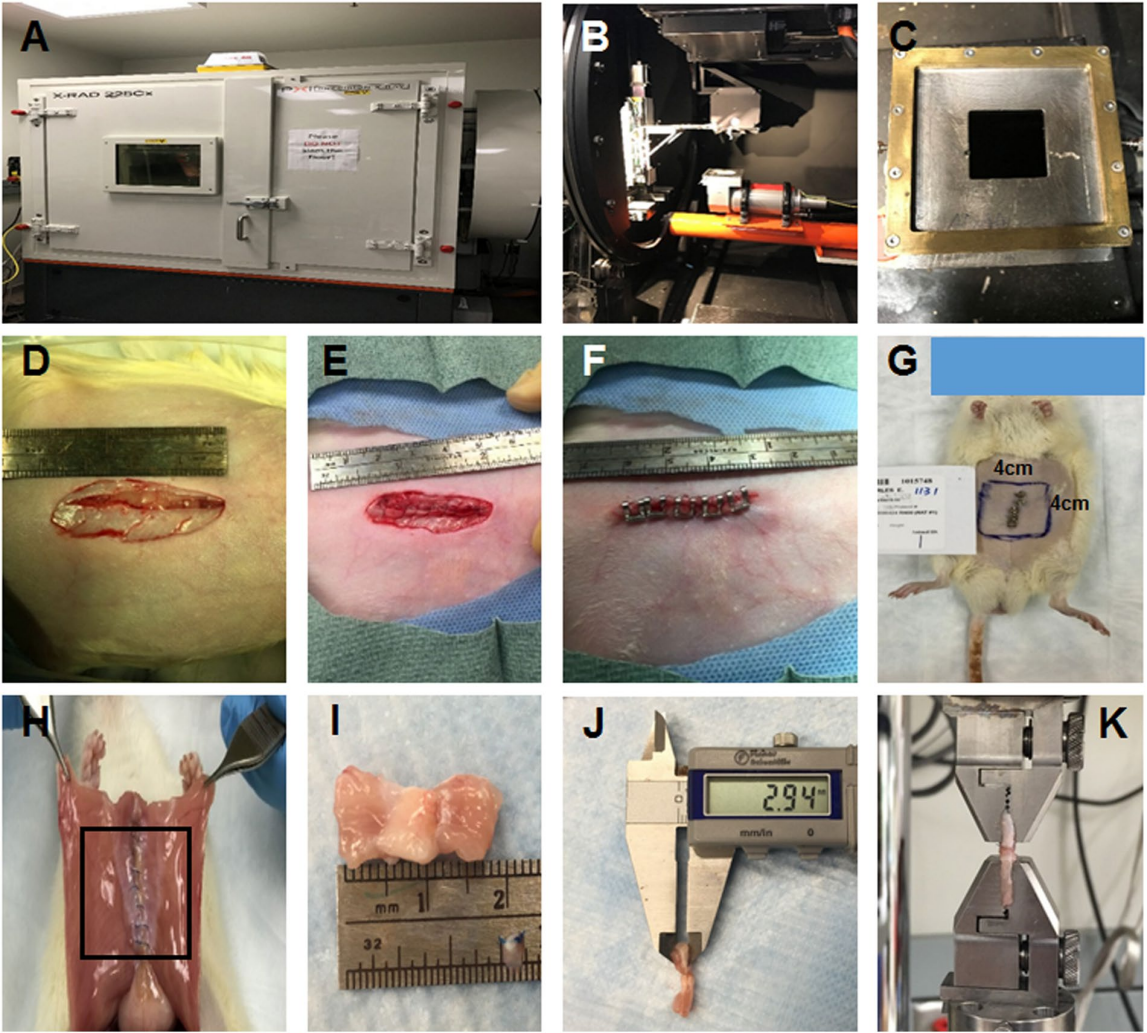
Rat irradiation, laparotomy, and muscle sample harvest. (**A**) XRAD225Xc radiotherapy unit setup. (**B**) Inside view of the XRAD225Xc. (**C**) The rat’s abdomen was covered with lead shield with 4 × 4-cm window on abdomen area. (**D**) Four weeks after radiation, a 3-cm laparotomy incision was made through the rat’s midline. (**E**) The rat’s laparotomy defect was repaired with running 5-0 Prolene sutures. (**F**) The skin wound was closed with a continuous intradermal suture using 6-0 Vicryl sutures. Skin staples were used to protect the repaired incision. (**G**) The 4 × 4 cm irradiated area of rat’s abdomen (blue box). (**H**) Two weeks after laparotomy, the irradiated abdominal wall area (black box) was surgically elevated to assess adhesions. (**I**) A 2 × 3-cm sample of the abdominal wall, including the repaired laparotomy incision, was harvested. The muscle sample was trimmed to 1 cm width × 2 cm length. (**J**) The thickness of muscle strip was measured using calipers. (**K**) The muscle was installed with a distance of 5 mm between the 2 clamps of mechanical tester.

The general strategy for radiation, surgery, and sample harvest is illustrated in Fig. 7. For radiation treatment, the rats were anesthetized with isoflurane in oxygen. The anesthetized rats were secured to a platform in a prone position. Lead shields were placed to protect other parts of the body from radiation (Fig. 7A–C). Rats were grouped according to the irradiation does (0-Gy, 10-Gy, and 20-Gy group) with 6 rats in each group. Radiation was administered by using the Precision XRAD 225Cx (Precision, North Branford, CT) with X-ray setting to 225kvp, 13 mA, cone 4 × 4 cm, beam time 267.78 s for 10-Gy treatment, while twice 10-Gy administration for 20-Gy treatment.

Four weeks after radiation, the rats underwent midline laparotomy under general anesthesia via inhaled isoflurane. A 3-cm incision was made along the midline through the skin, subcutaneous tissue, and linea alba (Fig. 7D). The laparotomy was then closed with running 5-0 Prolene (Ethicon, Somerville, New Jersey, USA) sutures, and the skin was closed with a continuous intradermal 6-0 Vicryl sutures (Ethicon, Somerville, New Jersey, USA) (Fig. 7E). Skin staples (Proximate, Ethicon) were used to reinforce the skin incision (Fig. 7F). After surgery, the rats were housed under the same conditions as before surgery but given antibiotics and analgesics (2.2 mg/ml Baytril (enrofloxacin) in water bottle for 3 days post-operation) and analgesic (0.01–0.05 mg/kg Buprenorphine subcutaneous injection, q12hours for 12–72 hours). The rats were assessed every day following the radiation and surgical treatment for the systemic and local complications, including weight loss, epilation, desquamation, erythema, blistering, swelling, hemorrhage, ulceration, necrosis, and dehiscence.

Two weeks after laparotomy, rats were euthanized with CO_2_ asphyxiation. The entire abdominal wall including the irradiated area was surgically excised to expose the abdominal cavity (Fig. 7G). The size (percentage of reconstructed area), type (omentum and intestine), and strength (grade) of adhesions were recorded (Fig. 7H). Adhesion strength was graded using the established Butler Adhesion Scale^38^. Zero represents no adhesion, 1 represents adhesions freed easily with gentle tension, 2 represents adhesions freed with blunt dissection, and 3 represents adhesions requiring sharp dissection. The full-thickness sample of the irradiated abdominal wall, including the incision site, was harvested. The skin was removed, the Prolene sutures were removed and the underlying musculofascial tissue was subjected to mechanical tests, histochemical and immunohistochemical analyses.

### Mechanical tests

Each muscle sample was trimmed to a 2-cm long, 1-cm wide with the incision in the middle and kept in Phosphate Buffered Saline (PBS) until testing. Mechanical testing was performed within 4 hours of sample harvest. Two muscle strips were analyzed per rat. The thickness of the muscle sample was measured (Fig. 7I,J). The mechanical properties of the muscle samples were determined by uniaxial tensile testing using the ElectroForce 3200 mechanical tester (Bose, Eden Prairie, MN, USA) with a load cell capacity of 250 N and a strain rate of 500 per second at room temperature. The muscle was installed tension-free with a distance of 5 mm between the two clamps of mechanical tester (Fig. 7K). Load and sample displacements were recorded. Stress is the load per unit area applied to the muscle sample, and strain is the ratio of elongation to the original sample length. They were calculated using the following formulas:

$$Stress=\frac{{\rm{Load}}}{{\rm{Width}}\times {\rm{Thickness}}}$$

$$Strain=\frac{{\rm{Displacement}}}{{\rm{Length}}}$$

Stress-strain curve of each sample was obtained based on the calculated stress and strain. The maximum strain (MS) is defined as the change in length when muscle sample fractured. The ultimate tensile stress (UTS) is the maximum tension a muscle sample can resist, which is measured as the peak point of the stress-strain curve. Once the applied load exceeded this value, the muscle sample became deformed until the incision separated. The elastic modulus (EM) is a measurement of the muscle’s resistance to elastic deformation caused by tensile force and is measured as the slope of the stress-strain curve in the linear region.

### Histochemical and immunohistochemical analyses

Harvested tissue samples were fixed in 4% paraformaldehyde, embedded in paraffin, and sliced into 5-mm-thick sections. The sections were deparaffinized, rehydrated, washed in distilled water, and mounted on slides. The slides were subjected to histological staining with hematoxylin and eosin (H&E) and Masson’s trichrome stain. For immunohistochemical staining, the slides were placed in heat-induced antigen retrieval citrate buffer (Biogenex, Fremont, CA) in a steamer at 95 °C for 10 min. Endogenous peroxidases were blocked by incubation with peroxide block (Innogenex, San Ramon, CA, USA), and nonspecific binding was blocked with normal goat serum (Vector Laboratories, Burlingame, CA). Sections were incubated with the primary antibody (1:200 anti-CD31; 1:200 anti-TGFβ; 1:200 anti- MYOD1; 1:400 anti-KI67; all from Abcam, Cambridge, MA) at 4 °C overnight. The sections were washed, and a biotinylated secondary antibody was applied for 30 min. The sections were then treated with streptavidin-horseradish peroxidase conjugate (Vectastain ABC kit, Vector Laboratories) and diaminobenzidine solution (DAB kit, Vector Laboratories) and then counterstained with hematoxylin. The sections were dehydrated, mounted, and imaged microscopically using a Vectra system (Perkin Elmer, Waltham, MA, USA). Histomorphometric analysis was performed with ImageJ 1.50c software (National Institutes of Health, Bethesda, MD, USA). The number of CD31, MYOD1, and KI67 positive cells were counted using an IHC toolbox plugin developed by Shu, *et al*.^39^. Microvessel density was determined by the counting distinct lumen-containing vessels consisting of CD31-positive vascular endothelial cells per HPF.

### Statistical analysis

Continuous data were expressed as mean ± standard deviation, or median and range if it was not normally distributed. Categorical data were reported as proportion or percentage. One-way analysis of variance (ANOVA) was performed to compare differences among 0-, 10-, and 20-Gy groups. Bonferroni correction was used for multiple comparison. A p-value < 0.05 was considered statistically significant. Statistical analysis was done using STATA v15.0 (StataCorp LLC, College Station, Texas, USA).
